# Ecological and health impacts of tobacco farming in Pakistan: A mixed-methods approach toward a sustainable pathway for agricultural transition

**DOI:** 10.18332/tid/201406

**Published:** 2025-03-26

**Authors:** Abdul Rasool Khoso, Gu Jintu, Qingjun Chen, Muhammad Javed Sheikh, Wang Suyuhan, Shahnaz Bhutto

**Affiliations:** 1 Department of Sociology, School of Public Administration, Hohai University, Nanjing, China; 2 Research Center for Environment and Society, Hohai University, Nanjing, China; 3 Hohai University, Nanjing, China; 4 Nanjing Polytechnic Institute, Nanjing, China; 5 Department of Rural Sociology, Faculty of Agricultural Social Sciences, Sindh Agriculture University, Tandojam, Pakistan

**Keywords:** tobacco farming, environmental degradation, health risks, sustainable agriculture, Pakistan

## Abstract

**INTRODUCTION:**

Tobacco farming plays a crucial role in the livelihoods of many rural communities in Pakistan, particularly in Khyber Pakhtunkhwa (KPK). However, this agricultural practice is associated with severe environmental degradation and significant health risks to workers during cropping.

**METHODS:**

This study evaluates the ecological and health impacts of tobacco farming in Pakistan, employing both quantitative (surveys) including 200 respondents (farmers and field workers/laborers) and qualitative methods (in-depth interviews) involving 10 respondents (farmers, policy experts, agriculturist and environmental specialists). The research focuses on Swabi, a key tobacco-growing region, and highlights the negative effects of excessive pesticide use, fertilizer application, and deforestation, which contribute to soil erosion, water pollution, and biodiversity loss

**RESULTS:**

Regression analysis shows that pesticide use (β=0.65, p<0.001) and deforestation (β=0.82, p<0.001) are the leading contributors to ecological degradation. The relationship between tobacco yield and environmental degradation, although showing a trend (p=0.062), is statistically negligible and unlikely to have practical significance (β= -0.15). Health risks are equally concerning, with farmworkers (labor hired for farming, farmers, landlords) exposed to harmful agrochemicals and nicotine absorption leading to respiratory diseases, skin conditions, and green tobacco sickness (GTS). Pesticide exposure (β=0.71, p<0.001) and contact with tobacco leaves (β=0.53, p<0.001) significantly impact workers' health, while using personal protective equipment (PPE) helps mitigate these risks (β= -0.43, p=0.001). The study also reveals that many farmers are interested in transitioning to alternative crops like maize or cotton, but they face financial and informational barriers.

**CONCLUSIONS:**

The growing of tobacco in Pakistan entails significant ecological and health dangers, emphasizing the immediate need for the implementation of sustainable farming strategies to mitigate environmental harm and enhance the socio-economic conditions of farmers. Government support through financial incentives, educational programs, and sustainable farming techniques is essential to reduce the environmental damage and improve public health.

## INTRODUCTION

Tobacco farming is an important agricultural activity in Pakistan, particularly in regions like Khyber Pakhtunkhwa (KPK) and Punjab, where it supports the livelihoods of many rural farmers^1^. The tobacco industry generates substantial tax revenue, with cigarette production being a major contributor. In 2020, tobacco contributed 0.5–1% of Pakistan’s GDP, with significant input from agriculture, manufacturing, and tax revenue^2^. Approximately 2 million people are involved in tobacco farming, including smallholder farmers and workers in processing and distribution^3^. Despite the economic benefits, tobacco cultivation imposes significant environmental and health challenges^4^. Tobacco farming contributes to deforestation, depletes soil nutrients, and relies heavily on pesticides and fertilizers, causing harm to local ecosystems. This can lead to soil degradation and decreased long-term agricultural productivity^5^. Additionally, health risks faced by farmers and laborers are often overlooked, with exposure to pesticides and nicotine absorption through the skin posing serious health concerns such as respiratory issues and skin problems^6^. These challenges underscore the need to critically assess tobacco farming’s impact on both ecological sustainability and public health^7^.

Tobacco farming poses severe environmental and health risks in various low-middle- income countries^8^. For example, in Brazil, large-scale tobacco cultivation contributes to deforestation, soil erosion, and water pollution, leading to significant biodiversity loss^9^. In Malawi, the intensive use of chemical fertilizers and pesticides for tobacco farming has resulted in soil degradation, which further worsens land productivity^10^. These chemicals not only harm the environment but also expose farmers and local communities to serious health risks, including respiratory issues and pesticide-related illnesses^11^. In India, tobacco curing methods often involve the burning of wood, which contributes to deforestation and air pollution, impacting both ecosystems and human health^12^. Groundwater contamination from the heavy use of fertilizers and pesticides is another significant concern. Zimbabwe also faces environmental degradation due to agrochemical use in tobacco farming, which has led to water pollution and health problems, such as increased risks of certain diseases in farming communities^13^.

Like other countries, Pakistan faces environmental degradation, soil erosion, and health risks among workers due to over-reliance on harmful chemicals. Deforestation, soil erosion, and overuse of harmful chemicals threaten the country’s ecological balance, while exposure to toxic chemicals affects the health of farmers^14^. Moving towards sustainable agricultural practices, such as crop diversification and the reduction of chemical inputs, can help reduce these negative impacts and promote environmental and public health^15^.

The environmental impacts of tobacco farming are both significant and varied. One major issue is deforestation, as land is cleared to grow tobacco, particularly in areas where land is already limited for other crops, such as KPK^16^. This deforestation results in habitat destruction, loss of biodiversity, and increased greenhouse gas emissions, contributing to climate change^17^. Tobacco farming also heavily depletes the soil, as the crop requires a large amount of nutrients^18^. This forces farmers to rely on synthetic fertilizers, which further degrade the soil over time, reducing its long-term productivity^19^. In addition, tobacco farming relies on large amounts of pesticides and herbicides to control pests and diseases. This leads to the contamination of soil and water, negatively affecting ecosystems and local communities^20^. In regions of Pakistan where access to clean water is already limited, the runoff from chemical treatments used on tobacco crops can have serious consequences for both aquatic life and human health.

The health risks posed by tobacco farming are also considerable. Farmers and laborers are exposed to harmful chemicals through the use of pesticides, herbicides, and fertilizers^21^. Prolonged exposure to these substances has been linked to various health issues, including respiratory diseases, skin conditions, and even cancer^22^. Another significant health concern is ‘green tobacco sickness’ (GTS), a type of nicotine poisoning that affects workers who handle the leaves without protection, causing symptoms like nausea, vomiting, and dizziness^23^. In Pakistan, the lack of proper protective gear exacerbates these health risks. Many small farmers and laborers, including women and children, do not fully understand the dangers of prolonged exposure to tobacco plants and the chemicals used in their cultivation. As a result, they experience chronic health issues, decreased productivity, and increased healthcare costs, which perpetuate poverty in regions that depend heavily on tobacco farming^22^.

Given the severe environmental and health challenges posed by tobacco farming, it is essential for Pakistan to explore sustainable agricultural alternatives. Transitioning to crops that are less harmful to the environment and pose fewer health risks could greatly benefit farmers^24^. Crops such as wheat, maize, or cash crops like cotton and sugarcane could provide viable economic alternatives while minimizing environmental damage^25^. Educating farmers about sustainable farming methods and providing them with access to resources like organic fertilizers and integrated pest management systems could reduce the ecological damage caused by tobacco farming^26^. Additionally, government incentives and investments in infrastructure could encourage farmers to make this transition, ensuring a more sustainable future for Pakistan’s agricultural sector.

Tobacco farming in Pakistan presents considerable environmental and health challenges. The heavy reliance on chemical pesticides and fertilizers in tobacco cultivation contributes to soil degradation, water pollution, and a decline in biodiversity^27^, while deforestation linked to land clearing further intensifies ecological damage^28^. Additionally, exposure to harmful agrochemicals negatively impacts the health of farmers and nearby populations, leading to respiratory problems and chronic health conditions. Despite these issues, the economic reliance on tobacco farming impedes efforts toward more sustainable agricultural practices. This study aims to investigate the need for a transition from tobacco farming to sustainable agricultural alternatives that mitigate environmental degradation and improve the health and well-being of farming communities in Pakistan.

## METHODS

This study employs a mixed-methods exploratory approach, combining both quantitative and qualitative techniques to evaluate the ecological and health impacts of tobacco farming in Pakistan, focusing on sustainable agricultural transitions. The primary objective is to assess the environmental degradation caused by tobacco cultivation, the health risks to farmworkers, and the potential for shifting to more sustainable farming practices.

### Study area and sample selection

The research was conducted in key tobacco-producing regions of Khyber Pakhtunkhwa (KPK), specifically focusing on Swabi, due to its heavy reliance on tobacco farming and the well-documented environmental and health challenges associated with the practice. Swabi is an ideal case study for tobacco farming in Pakistan due to its significant tobacco cultivation, its environmental and health challenges, and its representation of rural agricultural communities^29^. The sample size (n=200) was determined based on practical feasibility and guidelines for regression analysis, which recommend a minimum of 10–15 cases per independent variable. No power calculations were involved in the study.

### Ecological degradation index

An ecological degradation index was developed to quantify the environmental impacts of tobacco farming. The index provided a comprehensive measure of the ecological degradation occurring in the study areas, helping to quantify the environmental impact of tobacco farming practices. The index was calculated using weighted scores for pesticide usage, fertilizer application, deforestation rates, and tobacco yield. Data for these variables were collected through field observations and expert consultations, and each factor was assigned a weighted score to reflect its contribution to environmental harm. Weights were assigned based on expert consultations, with pesticide usage receiving the highest weight due to its significant ecological impact^30^. Similarly, the health status index was based on self-reported symptoms of respiratory illness, skin conditions, and GTS, with scores normalized on a 0–1 scale. Both indices were treated as continuous variables for regression modeling.

### Quantitative data collection

*Health data collection*

Structured interviews and health surveys were conducted with a sample of 200 farmworkers (farmers, labors working in the tobacco field) to understand the health risks associated with tobacco farming. Key variables included:

- Pesticide exposure: measured by the number of hours per week spent applying or working near pesticide-treated fields.
- Exposure to tobacco leaves: measured in hours per week of contact with fresh tobacco leaves.
- Total working hours per week: collected to assess the overall labor demands on workers.
- Access to personal protective equipment (PPE): a binary variable indicating whether workers were provided with PPE while handling chemicals or tobacco plants (0=no, 1=yes).

*Regression analysis*

Multiple linear regression was used to explore the relationships between tobacco farming practices and their ecological and health impacts. For ecological degradation, the model is:

Y_ecological_ = β_0_ + β_1_ X_1_ (pesticides) + β_2_ X_2_ (fertilizer) + β3 X3(deforestation) + β4 X4 (tobacco yield) + ϵ

where Y_ecological_ is the ecological degradation index (continuous variable), the β are regression coefficients, and ϵ is residual error. Similarly, the health outcome index is:

Y_health_ = β_0_+ β_1_ X_1_ (pesticide exposure) + β_2_ X_2_ (tobacco exposure) + β_3_ X_3_ (working hours) + β_4_ X_4_ (PPE) + ϵ

These models were assessed for statistical significance to identify the primary factors associated ecological degradation and health risks in tobacco farming communities. To address potential multicollinearity and spurious associations, variance inflation factor (VIF) analysis was performed. Independent variables with VIF >5 were excluded or centered to reduce collinearity. Independent variables were selected based on biological plausibility, prior literature, and relevance to the study objectives. For instance, pesticide and fertilizer usage were included due to their well-documented environmental impacts, while deforestation rate and tobacco yield were selected to capture broader ecological and economic effects. Linear regression identifies associations between predictors and outcomes but does not imply causation given the cross-sectional design.

### Qualitative data collection

*Farmer and worker interviews*

To supplement the quantitative data, in-depth interviews were conducted with 10 participants (5 farmers, 5 experts), selected through purposive sampling to represent a range of farm sizes and regions covering, Perceptions were assessed on soil quality and environmental changes over time, health issues related to the application of pesticide and tobacco plants exposure, and challenges and willingness to transition to alternative crops. The local farmers, farm workers were recruited during their time to crop, which met the research outcomes easily for interviews.

*Expert consultations*

Consultations with agricultural experts (n=2), policymakers (n=2), and environmental specialists (n=1) provided additional insights into the ecological effects of tobacco farming and the potential for adopting more sustainable agricultural practices. These consultations helped inform the study’s recommendations for future agricultural transitions. However, the data from respondents provided rich information and the limit of 10 respondents were to gather sufficient knowledge for the study. The researchers approached the experts through extension officers, the Public Policy Analysis department and the National Tobacco Control department.

### Statistical analysis

The statistical analysis for this study was conducted using IBM SPSS Statistics 27.0. Regression models were examined for multicollinearity, heteroscedasticity, and overall fit to ensure accuracy and validity^31^. Key statistical metrics such as R^2^, t-values, and p-values were calculated for each variable. Multicollinearity among independent variables was assessed using the variance inflation factor (VIF). Variables with VIF >5 were excluded from the model. All statistical tests conducted in this study were two-tailed, with a significance threshold set at p<0.05.

### Qualitative analysis

The qualitative data, collected through in-depth interviews with farmers, farm workers, and experts, were analyzed using NVivo software and a thematic analysis approach. Key themes such as soil degradation, health effects (respiratory issues, GTS), and the potential for sustainable farming were identified, providing context to the quantitative findings. The coding process followed an inductive approach, with two independent coders coding a subset of transcripts. Discrepancies were resolved through discussion and consensus to ensure reliability. A final set of themes was developed, linking them to the quantitative data to enhance understanding of the ecological and health impacts of tobacco farming. Interviews were conducted orally with the help of a translator, ensuring accurate communication. Data were recorded, translated, transcribed, and coded. The data collection process was from 10 December 2023 to 20 January 2024, to collect the survey questionnaires and conduct the in-depth interviews.

### Ethical considerations

Ethical review and approval were waived for this study on the ecological and health impacts of tobacco farming in Pakistan, as it primarily focuses on secondary data analysis and publicly available information. The study did not involve minors, vulnerable populations, or invasive procedures, and adheres to ethical standards outlined in the 1964 Helsinki Declaration and subsequent amendments. No identifiable personal information was collected, and the research poses minimal risk to participants, comparable to daily life activities. The Ethics Review Committee of the School of Public Administration, Hohai University determined that the study qualifies for exemption from ethical review based on these factors. Informed consent was obtained where necessary, with participants fully aware of the study’s objectives and their voluntary involvement.

### Reliability and validity of the data

Table 1 shows the reliability and validity results for three variables: ecological impacts, health risks, and sustainable agriculture. Reliability is measured by Cronbach’s α, which reflects internal consistency. Ecological impacts have an α=0.68 in the pre-test (n=25) and 0.71 in the final test (n=200), indicating moderate consistency. Health risks display higher reliability with values of 0.71 and 0.73, while sustainable agriculture improves from 0.68 to 0.72 between the two tests. The average variance extracted (AVE) values demonstrate acceptable validity, with all variables meeting the threshold of 0.40, and health risks achieving a high AVE of 0.71, indicating strong validity. Overall, the analysis confirms that the measures used are reliable and valid for the study.

**Table 1:** Reliability and validity of the data

*Variable*	*Items*	*Cronbach’s alpha*		*CFA*
		*Pre-test (N=25)*	*Final test (N=200)*	*AVE*
Ecological impacts	7	0.68	0.71	0.48
Health risks	8	0.71	0.73	0.71
Sustainable agriculture	7	0.68	0.72	0.58

## RESULTS

### Descriptive indicators of the respondents

Table 2 summarizes the demographic information of the respondents including age, education level, occupation, farming experience, and income. Age of respondents ranged from 28 to 58 years, with a mean of 46 years (SD=9.33) during the field survey, targeting male respondents due to the nature of the study and male dominant society. Most respondents had primary education (34%), while a smaller portion had higher degrees, such as Bachelor’s or Master’s (14.5%). Farming is the predominant occupation, with nearly half of the respondents engaged in it (48%), followed by landlords (29%), self-employed individuals (12.5%), and those in government jobs (10.5%). In- terms of farming experience, the majority (43.5%) had 4–7 years’ experience, with fewer respondents being either newcomers or highly experienced. Overall, the data reflect a group with strong ties to agriculture and varying levels of education and farming expertise. Income ranged from 200000 to 1200000 PKR (1000 Pakistani Rupees about US$3.5), and the average income was 750000 PKR.

**Table 2:** General characteristics of the respondents (N=200)

*Characteristics*	*n*	*%*
**Age** (years), mean (SD)	46.00	9.33
**Seasonal income** (PKR), mean (SD)	750000	331976
**Education level**		
Illiterate	32	16.0
Primary	68	34.0
Intermediate	37	18.5
Bachelor’s/undergraduate	34	17.0
Master’s or higher	29	14.5
**Occupation**		
Farmer	96	48.0
Landlord	58	29.0
Self-business	25	12.5
Government job	21	10.5
**Farming experience** (years)		
1–3	39	19.5
4–7	87	43.5
8–9	43	21.5
>9	31	15.5

### Regression model

Most variables demonstrated significant relationships; however, the relationship between tobacco yield and ecological degradation was found to be non-significant. This suggests that while tobacco yield contributes to the economic aspect of farming, its direct influence on environmental degradation may not be as substantial compared to other factors like pesticide and fertilizer use or deforestation. This finding indicates that addressing yield alone may not be sufficient for mitigating environmental harm, emphasizing the need to focus on other agricultural practices with more direct ecological consequences.

In evaluating the ecological and health impacts of tobacco farming in Pakistan, a multiple linear regression analysis was performed to understand the relationship between farming practices (such as pesticide use, fertilizer application, deforestation, and tobacco yield) and two key outcomes: 1) ecological degradation; and 2) health outcomes for workers.

Table 3 presents a statistical analysis examining the effects of four independent variables: pesticide usage, fertilizer usage, deforestation rate, and tobacco yield, on a dependent variable. The coefficient (β) shows that pesticide usage (0.65), fertilizer usage (0.47), and deforestation rate (0.82) have positive relationships with the dependent variable, suggesting that increases in these factors lead to increases in the dependent variable. In contrast, tobacco yield (-0.15) has a negative relationship, indicating a potential decrease in the dependent variable with higher tobacco yield. The standard errors for all variables are relatively low, showing that the estimates are precise. The t-values for pesticide usage (5.42), fertilizer usage (5.22), and deforestation rate (7.45) are high, supporting the statistical significance of their effects, with corresponding p-values all less than 0.001. However, the p-value for tobacco yield is 0.062, indicating that its relationship is not statistically significant at the 5% level.

**Table 3:** Regression analysis of ecological degradation factors and tobacco yield

*Variables*	*Coefficient (β)*	*SE*	*t-value*	*p*
Pesticide usage (kg/hectare)	0.65	0.12	5.42	<0.001
Fertilizer usage (kg/hectare)	0.47	0.09	5.22	<0.001
Deforestation rate (hectares/year)	0.82	0.11	7.45	<0.001
Tobacco yield (kg/hectare)	-0.15	0.08	-1.88	0.062

Ecological degradation was assessed using a composite index incorporating soil fertility loss, contamination of water sources (due to pesticide runoff), and deforestation rates. SE: standard error.

### Health outcomes based on pesticide and tobacco exposure

**Figure 1:**
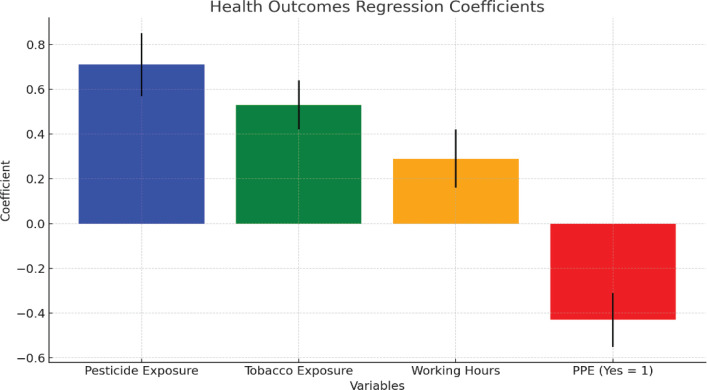
Health outcomes regression coefficients

To explore health outcomes, a similar regression model was applied to assess how workers’ health was impacted by: pesticide exposure (hours/week); exposure to tobacco leaves (hours/week, to capture GTS); working hours per week; and access to personal protective equipment (PPE) (binary variable: 0=no, 1=yes). Figure 1 illustrates the regression coefficients for health outcomes. Pesticide exposure has the highest positive coefficient at approximately 0.7, followed by tobacco exposure at 0.5, and working hours at 0.3, indicating a direct relationship between these variables and worsening health outcomes. PPE usage shows a negative coefficient around -0.3, suggesting it helps mitigate negative health effects. The black lines on each bar represent the confidence intervals for these estimates.

### Correlation analysis

To provide further insights, correlation analyses were performed to examine the relationship between pesticide exposure and health outcomes. The results indicated strong positive correlations between pesticide use and health issues among farmworkers, including higher incidences of GTS and other illnesses linked to pesticide exposure. A correlation analysis was also conducted to investigate the relationship between smoking frequency and various demographic and health factors.

Table 4 presents a correlation analysis that evaluates the relationships between pesticide exposure, tobacco exposure, working hours, and the use of personal protective equipment (PPE). The results show a moderate positive correlation between pesticide exposure and tobacco exposure (r=0.617), indicating that individuals who are exposed to pesticides are somewhat more likely to also be exposed to tobacco. There is a moderate positive correlation between pesticide exposure and working hours (r=0.411), suggesting that longer working hours are somewhat associated with higher pesticide exposure. A similar positive correlation is observed between tobacco exposure and working hours (r=0.461), implying that individuals who work longer hours are also more likely to be exposed to tobacco. The use of PPE, however, is negatively correlated with all three variables: pesticide exposure (r= -0.208), tobacco exposure (r= -0.103), and working hours (r= -0.163), suggesting that those who use PPE tend to have lower levels of exposure to pesticides, tobacco, and work fewer hours. These findings highlight the complex relationships between work-related exposures and protective measures in this population.

**Table 4:** Correlation analysis assessing the relationship between pesticide exposure and health outcomes

	*Pesticide exposure*	*Tobacco exposure*	*Working hours*	*PPE usage*
Pesticide exposure	1			
Tobacco exposure	0.61656	1		
Working hours	0.411418	0.46145	1	
PPE usage	-0.20814	-0.10293	-0.16278	1

Pearson’s and Spearman’s correlation coefficients were calculated to assess the relationships^32^.

### Knowledge of local farmers about sustainable agriculture

Table 5 presents data from 200 participants who rated various statements related to sustainable agriculture and the effects of tobacco farming using 5-point Likert scale. The mean scores, ranging 3.3–4.8, indicate a general consensus in agreement with the statements. The highest average score (4.8) was observed for the statement ‘Tobacco farming harms worker health’, indicating strong agreement, while the lowest mean score (3.3) was for ‘Switching to alternative crops is financially viable’, showing more diverse opinions. Standard deviation values range from 0.2 to 0.9, with lower variability for health-related statements (SD=0.2) and government support for alternative crops (SD=0.3), suggesting consistent responses in these areas. The statement regarding the financial feasibility of switching to alternative crops had the highest variability (SD=0.9), pointing to more mixed views. Overall, the responses highlight significant support for sustainable agriculture, the need for government intervention, and an acknowledgment of the harmful effects of tobacco farming on health and the environment.

**Table 5:** Perceptions of local farmers regarding sustainable agriculture (N=200)

*Statement*	*Min*	*Max*	*Mean*	*SD*
Sustainable agriculture is more eco-friendly	3	5	4.2	0.6
Transitioning from tobacco farming is necessary.	2	5	4.1	0.8
The government should support alternative crops	4	5	4.5	0.3
Sustainable practices improve long-term yield	3	5	4.0	0.7
Switching to alternative crops is financially viable	2	4	3.3	0.9
Tobacco farming harms worker health	4	5	4.8	0.2
Tobacco farming leads to environmental degradation	4	5	4.7	0.3
Sustainable farming improves community well-being	3	5	4.0	0.7
Agrochemicals are harmful to soil health	4	5	4.6	0.4
The use of protective equipment reduces health risks	3	5	4.2	0.5

### Regression analysis of health outcomes based on pesticide and tobacco exposure

Regression analysis of health outcomes based on pesticide and tobacco exposure found that higher pesticide exposure dramatically increases the risk of negative health outcomes such as respiratory and skin conditions (β=0.71, p<0.001) and that deforestation (β=0.82, p<0.001) is a major contributor to ecological deterioration among tobacco farmworkers in Pakistan (Table 6).

**Table 6:** Regression analysis of health outcomes based on pesticide and tobacco exposure

*Variables*	*Coefficient (β)*	*SE*	*t-value*	*p*
Pesticide exposure	0.71	0.14	5.07	<0.001
Tobacco exposure	0.53	0.11	4.82	<0.001
Working hours	0.29	0.13	2.23	0.028
PPE usage	-0.43	0.12	-3.58	0.001

SE: standard error.

### Challenges of tobacco farming: Ecological, health, and transition issues

**Figure 2:**
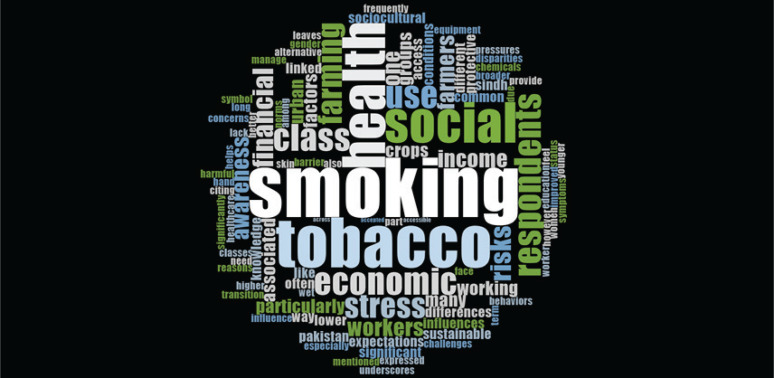
Word cloud for ecological impacts, health risks and agricultural transitions

The word cloud (Figure 2) visually represents the key themes from the study. The larger words show the most frequently used words. The analysis was done thematically and the results were generated using NVIVO 15.exe.

*Ecological impacts of tobacco farming*

Farmers in Khyber Pakhtunkhwa (KPK) are increasingly worried about the long-term sustainability of tobacco cultivation. Many farmers have noticed a gradual decline in soil fertility, with one farmer mentioning:

*‘The more fertilizers we apply, the less productive the land becomes.’*

This comment highlights the diminishing returns from continued use of chemical inputs, aligning with research that indicates nutrient depletion in soils where tobacco is grown. The environmental damage extends beyond soil health, as tobacco farming in regions like Swabi significantly contributes to deforestation. Clearing land for tobacco cultivation is a major cause of biodiversity loss and ecosystem disruption, according to environmental experts.

Pesticide use is another growing concern. Farmers report that pest resistance has led to a notable increase in pesticide application. One farmer shared:

*‘We have to use more chemicals now because the pests no longer respond to the usual methods, and it’s affecting our crops and water supply.’*

Environmental advocates warn that runoff from these farms is contaminating nearby water sources, adversely affecting both local wildlife and communities that rely on the water for drinking and agriculture.

*Health risks associated with tobacco farming*

Health risks linked to tobacco farming are well-documented by the workers involved. Many workers experience symptoms associated with GTS, including dizziness, nausea, and skin irritation, particularly after handling wet tobacco leaves. One worker said:

*‘Whenever I work with the wet leaves, I feel sick, and the symptoms can last for hours.’*

There is a noticeable lack of protective gear, with many workers citing the high cost as a barrier to using gloves, masks, or other protective equipment. One worker mentioned:

*‘We know the chemicals are harmful, but we can’t afford the protection, and our employers don’t provide it.’*

Long-term exposure to pesticides also raises significant health concerns. Workers frequently report respiratory issues, chronic skin conditions, and other health problems. The increased use of chemicals due to pest resistance exacerbates these risks, placing farmworkers in hazardous conditions. This situation underscores the urgent need for better health and safety standards in tobacco farming, along with improved access to protective equipment and medical care.

*Sustainable agricultural transition*

Some farmers have expressed a desire to shift away from tobacco farming, but they face significant challenges in making this transition. Deficiencies of knowledge about alternative crops are concerned about the financial risks associated with changing their farming practices. As one farmer pointed out:

*‘We would like to grow crops like maize or cotton, but tobacco is the only one that gives us a stable income.’*

This reliance on tobacco farming poses a major barrier, as farmers are uncertain whether alternative crops will provide the same financial security. With regard to the experts’ opinions, one of the respondents suggested to advocate for government support to assist farmers in transitioning successfully. Agriculture experts recommended that the government should indorse educational programs to inform farmers about sustainable crop alternatives, financial incentives to reduce tobacco cultivation, and training on sustainable farming methods. These initiatives could encourage more farmers to switch to crops that are less harmful to the environment and pose fewer health risks to workers, promoting both environmental sustainability and improved livelihoods.

## DISCUSSION

The study highlights the considerable environmental and health challenges linked to tobacco farming in Pakistan, especially in Swabi district Khyber Pakhtunkhwa (KPK). Both quantitative and qualitative evidence demonstrates that tobacco cultivation contributes to significant environmental degradation and poses serious health risks to workers.

The regression analysis revealed that the primary drivers of environmental degradation are pesticide use, fertilizer application, and deforestation. These factors not only reduce soil quality but also contaminate water bodies through pesticide runoff, adversely affecting local ecosystems and communities. Previous research supports these findings, which emphasize the adverse environmental impacts of tobacco farming, including deforestation and soil nutrient depletion^33^. Additionally, many farmers in this study expressed concerns over the long-term fertility of their land and the increasing need for chemical inputs to maintain yields.

Health risks among tobacco farmworkers are also alarming. The data show that prolonged exposure to pesticides and tobacco leaves significantly contributes to negative health outcomes, with many workers reporting symptoms of GTS, respiratory issues, and skin conditions. These health risks are heightened by the lack of access to protective gear, as workers stated during interviews. These findings echo previous research, which underscores the occupational hazards associated with tobacco farming, particularly in low-resource settings where protective measures are limited^34^. According to the research, farmworkers who are exposed to higher levels of pesticides are at a significantly higher risk of developing respiratory ailments and green tobacco sickness. According to the analysis, the primary causes of environmental damage are the use of pesticides, fertilizers, and the destruction of forests. These methods not only damage the soil and water, but they also make it more difficult for landowners to grow crops that thrive in the future. While tobacco farming does not directly harm the environment, the substances and deforestation required to support it do. Simply put, the way tobacco products is farmed harms the environment and creates permanent difficulties for societies that rely on appropriate land for a living.

The study also uncovered several challenges that hinder farmers from moving away from tobacco farming toward more sustainable agricultural practices. While many farmers showed a willingness to transition to alternative crops, they identified financial instability, a lack of knowledge, and inadequate government support as significant barriers. This observation aligns with global research findings. For instance, studies in Bangladesh and Zimbabwe have revealed that similar obstacles impede efforts to reduce tobacco cultivation and embrace sustainable farming practices^27^. The farmers in this study stressed the need for financial incentives and training to support the shift to alternative crops like maize, wheat, or cotton, which are less harmful to the environment and offer better long-term economic potential. Research from Brazil has also highlighted the critical role of government programs that offer economic support and training to assist farmers in their transition away from tobacco cultivation^35^. Addressing these barriers is critical for promoting a shift to more sustainable agricultural practices. The study’s findings are consistent with the provisions of Article 18 of the Framework Convention on Tobacco Control (FCTC) that highlights safeguarding both the environment and public health from the negative consequences of tobacco farming and production^36^.

### Limitations

This study has several limitations that should be considered when interpreting the results. First, the sample size may be too small or not representative of the broader population, which limits the generalizability of the findings. Second, the study’s cross-sectional design restricts it to establishing associations rather than causality, which could be addressed in longitudinal studies. The use of self-reported data for variables such as tobacco exposure, working hours, and PPE usage introduces potential recall and social desirability bias. Despite controlling for some variables, the presence of unmeasured confounders (e.g. diet, socioeconomic status) may still influence the results. The study also has a limited scope of variables, as it may not account for all relevant health outcomes or exposures. Measurement errors related to pesticide exposure and PPE use could further affect the accuracy of the findings. Additionally, the research is limited by geographical and temporal constraints, meaning the findings may not apply universally across regions or time periods. Lastly, ethical issues may arise regarding privacy and informed consent, especially in studies involving sensitive health data.

## CONCLUSIONS

This study draws attention to the serious health and environmental risks associated with cultivation of tobacco in Pakistan, particularly in areas like Swabi. The results demonstrate that excessive use of fertilizers, pesticides, and deforestation are the main causes of environmental degradation, with tobacco yield having very little effect on the environment. Continuous exposure to such agricultural chemicals along with frequent interactions with tobacco leaves can have a serious negative impact on farmworkers’ health, including respiratory disorders and GTS. However, accessibility and application of safety gear has been shown to reduce these dangers to health. Farmers in the study conveyed an interest in switching to alternative crops such as maize or cotton, despite obstacles such as financial constraints, lack of expertise, and insufficient institutional support. Overall, while cultivating tobacco may generate short-term economic benefits, the harm to the environment and health risks necessitate an intentional move toward environmentally friendly agriculture. Growing tobacco in Pakistan entails significant ecological and health dangers, emphasizing the immediate need for the implementation of sustainable farming strategies to mitigate environmental harm and enhance the socio-economic conditions of farmers.

## Data Availability

The data supporting this research are available from the authors on reasonable request.
